# Non-Invasive Profiling of Advanced Prostate Cancer via Multi-Parametric Liquid Biopsy and Radiomic Analysis

**DOI:** 10.3390/ijms23052571

**Published:** 2022-02-25

**Authors:** Gareth Morrison, Jonathan Buckley, Dejerianne Ostrow, Bino Varghese, Steven Y. Cen, Jeffrey Werbin, Nolan Ericson, Alexander Cunha, Yi-Tsung Lu, Thaddeus George, Jeffrey Smith, David Quinn, Vinay Duddalwar, Timothy Triche, Amir Goldkorn

**Affiliations:** 1 Division of Medical Oncology, Department of Medicine and Department of Biochemistry & Molecular Medicine, Keck School of Medicine and Norris Comprehensive Cancer Center, University of Southern California, Los Angeles, CA 90089, USA; garethm82@gmail.com (G.M.); acunhatuna@gmail.com (A.C.); allnovels@gmail.com (Y.-T.L.); diquinn@med.usc.edu (D.Q.); 2 Department of Population and Public Health Sciences, Keck School of Medicine and Norris Comprehensive Cancer Center, University of Southern California, Los Angeles, CA 90089, USA; jbuckley@usc.edu; 3 Center for Personalized Medicine, Department of Pathology and Laboratory Medicine, Children’s Hospital Los Angeles, Los Angeles, CA 90027, USA; dostrow@chla.usc.edu (D.O.); triche@usc.edu (T.T.); 4 Department of Radiology, Keck School of Medicine and Norris Comprehensive Cancer Center, University of Southern California, Los Angeles, CA 90089, USA; bino.varghese@med.usc.edu; 5 Departments of Radiology and Neurology, Keck School of Medicine, University of Southern California, Los Angeles, CA 90089, USA; cen@usc.edu; 6 RareCyte, Inc., Seattle, WA 98121, USA; jwerbin@gmail.com (J.W.); nericson@rarecyte.com (N.E.); tgeorge@rarecyte.com (T.G.); 7 Clinical Sequencing Division, Thermo Fisher Scientific, San Francisco, CA 94080, USA; jeff.smith@thermofisher.com; 8 Departments of Radiology and Urology, Keck School of Medicine and Norris Comprehensive Cancer Center, University of Southern California, Los Angeles, CA 90089, USA; vinay.duddalwar@med.usc.edu

**Keywords:** metastatic prostate cancer, cell-free DNA, circulating tumor cells, radiomics

## Abstract

Integrating liquid biopsies of circulating tumor cells (CTCs) and cell-free DNA (cfDNA) with other minimally invasive measures may yield more comprehensive disease profiles. We evaluated the feasibility of concurrent cellular and molecular analysis of CTCs and cfDNA combined with radiomic analysis of CT scans from patients with metastatic castration-resistant PC (mCRPC). CTCs from 22 patients were enumerated, stained for PC-relevant markers, and clustered based on morphometric and immunofluorescent features using machine learning. DNA from single CTCs, matched cfDNA, and buffy coats was sequenced using a targeted amplicon cancer hotspot panel. Radiomic analysis was performed on bone metastases identified on CT scans from the same patients. CTCs were detected in 77% of patients and clustered reproducibly. cfDNA sequencing had high sensitivity (98.8%) for germline variants compared to WBC. Shared and unique somatic variants in PC-related genes were detected in cfDNA in 45% of patients (MAF > 0.1%) and in CTCs in 92% of patients (MAF > 10%). Radiomic analysis identified a signature that strongly correlated with CTC count and plasma cfDNA level. Integration of cellular, molecular, and radiomic data in a multi-parametric approach is feasible, yielding complementary profiles that may enable more comprehensive non-invasive disease modeling and prediction.

## 1. Introduction

Approval of novel therapies for treatment of advanced prostate cancer has accelerated at an unprecedented rate, improving clinical outcomes and quality of life [1,2,3,4,5]. In contrast, development of clinically validated biomarkers to guide the optimal sequence of therapy, provide real-time monitoring, or identify treatment resistance has lagged. Traditional tissue biopsies have limited utility in advanced metastatic disease [6], which has driven the field to consider alternative diagnostic tools such as radiomics [7] and liquid biopsies [8]. These technologies are repeatable, non-invasive or minimally invasive and hold the promise for discovering prognostic biomarkers, with a single blood draw enabling characterization of circulating tumor cells (CTCs), cell-free DNA and RNA, germline DNA and extracellular vesicles.

CTC count as a prognostic indicator of response in metastatic hormone-sensitive prostate cancer (mHSPC) and metastatic castration-resistant prostate cancer (mCRPC) treated with androgen receptor signaling inhibitors (ARSIs) has been validated prospectively in multiple clinical trials [9,10,11], and dynamic monitoring of CTC counts early during treatment demonstrated an association with clinical response [12,13,14,15]. More recently, CTC characterization studies through genomic, phenotypic and morphological profiling have yielded novel insights into the underlying tumor biology of advanced prostate cancer [16,17]. However, clinically validated CTC biomarkers are currently limited to androgen receptor splice variant 7 (ARv7)-positive CTC assays that identified patients who respond better to taxane chemotherapy versus ARSIs [18,19,20,21]. 

Elevated levels of baseline cell-free tumor DNA have been shown to correlate with worse clinical outcomes in mCRPC on ARSIs [22,23], and presence of androgen receptor copy number gains or somatic alterations was found to correlate with shorter progression-free survival (PFS) and overall survival (OS), providing a rationale for early treatment switching to chemotherapy or PARPi [24,25]. For monitoring treatment response, a reduction in cfDNA concentrations post-treatment has been shown to correlate with improved OS [26]. For patients who progress on targeted therapies, identification of mutations associated with putative resistance mechanisms has provided a rationale for monitoring for these alterations in future prospective trials. 

While analyzing liquid biopsy components individually has yielded important insights, to date, few studies have attempted to directly compare the genomic information gained from circulating cells versus cell-free material from the same blood sample. Such studies are critical to identifying which analytes provide the most disease-relevant and clinically actionable information. Moreover, integrating information from multiple liquid biopsy components has the potential to yield more comprehensive and accurate molecular profiles of the evolving disease and ultimately more effective biomarkers to guide therapy. Our group and others have begun to develop methods that can investigate multiple liquid biopsy components in parallel for a more comprehensive patient profile [17,27]. For example, workflows that can profile DNA alterations in both CTCs and cfDNA have the advantage of enabling tumor profiling in those patients without CTCs, providing greater confidence in calling shared alterations, and enabling investigations into the importance of mutations that are unique to either. Additionally, CTC characterization enables further evaluation of treatment-driven phenotypic and genomic heterogeneity that could identify emergence of resistance clones.

Another complementary, non-invasive approach for monitoring therapy in advanced prostate cancer is the emerging field of radiomic imaging analysis. Radiomic analysis is the extraction of quantitative imaging features from standard-of-care medical imaging such as computed tomography (CT) [28,29]. In prostate cancer, radiomic analysis has had wide-ranging utility, including tumor detection and localization, monitoring of treatment response, and prognostic analysis. Similarly, initial radiogenomic studies in prostate cancer have begun to investigate associations between these radiomic features and prognostic biomarkers with the aim of complementing genetic testing with imaging biomarkers [30,31]. There are multiple methods of incorporating radiomics in multi-center studies that allow for variation of imaging acquisition. These include phantom-based studies [32] and software-based harmonization techniques [33]. Integration of these innovative liquid biopsy and radiomic tools to monitor therapy response and emergence of treatment resistance has great potential to dramatically improve outcomes of prostate cancer patients.

In this cohort of advanced prostate cancer patients, we evaluated the feasibility of a multi-parametric, non-invasive profiling approach that integrates liquid biopsy readouts for CTC enumeration, CTC Arv7 and synaptophysin (SYP) staining, genomic analysis of individual CTCs and matched plasma cfDNA, and CTC clustering analysis together with comprehensive radiomic analysis of companion bone metastasis CT scans. We aimed to assess whether the liquid biopsy readouts described could be obtained using RareCyte’s CTC characterization technology and an identical sequencing panel to allow for direct comparison of cfDNA and CTC variants. In addition, we aimed to evaluate how non-invasive CT-based radiomic analysis correlated with CTC and cfDNA readouts from matched patients.

## 2. Results

### 2.1. Patient Cohorts

A summary chart of all patients’ clinical data can be found in Table S1. At time of blood draw, the 22 men with advanced metastatic prostate cancer recruited to our study for liquid biopsy and radiomic analysis had a median age of 72 years and a median PSA of 18.7 ng/mL; 8 of the 22 patients had presence of skeletal metastasis. Patients had undergone multiple lines of standard therapy (median number of 4 standard therapies) including 80% having received 2nd line ARSI, 50% having received chemotherapy, and 20% having received either radium-223 or sipuleucel-T. At time of blood draw, approximately 60% of patients exhibited progressive disease on current therapy. 

### 2.2. CTC Enumeration and Quantitative Immunofluorescence

**Figure 1:**
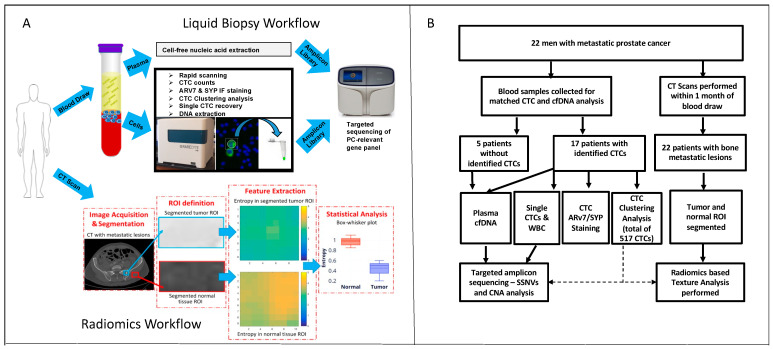
Multiparametric liquid biopsy workflow. (**A**). Peripheral blood is drawn and delivered onsite to the Liquid Biopsy Core, where it is separated into plasma and mononuclear cell components. Cell-free nucleic acid is extracted from plasma. CTCs and WBCs are identified using immunofluorescent staining and automated high content imaging. Single cells are recovered for DNA extraction. Plasma and single cell derived nucleic acids are used to generate amplicon libraries for targeted sequencing of PC-relevant genes. Matched CT scans undergo image segmentation, region of interest (ROI) definition, feature extraction, and statistical analysis. (**B**). Consort diagram outlining patients, samples, and workflows performed in this study.

**Figure 2:**
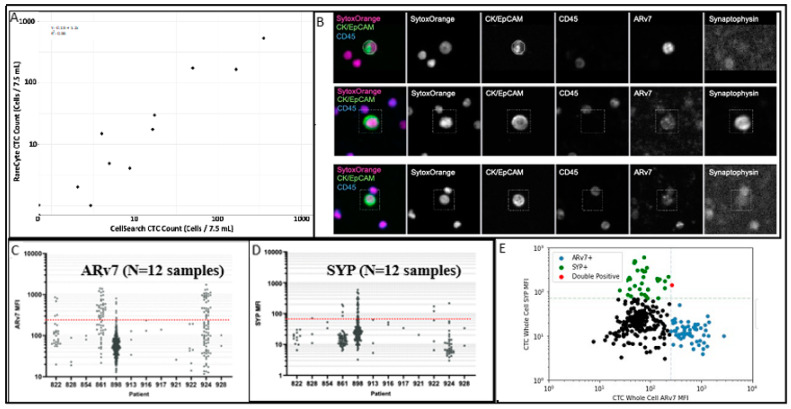
CTC enumeration and quantitative immunofluorescence. (**A**). CTC counts displayed as a comparison between Cell Search and RareCyte counts. (**B**). Staining profile of representative CTC with ARv7 positive staining (**top**); Synaptophysin positive staining (**middle**) and negative for both markers (**bottom**). (**C**). ARv7 MFI distribution for 284 CTCs from 12 prostate cancer patients. Red dotted line indicates the cut-off value for positivity (MFI = 250). (**D**). SYP MFI distribution for 284 CTCs from 12 prostate cancer patients. Red dotted line indicates the cut-off value for positivity (MFI = 70). (**E**). SYP vs. ARv7 MFI distribution for 284 CTCs from 12 prostate cancer patients.

The overall sample analysis workflow is summarized in Figure 1. All 22 patients in this study underwent RareCyte enumeration, and 17 of these (77%) had detectable CTCs, with a median of 1.5 CTCs/7.5 mL (range: 1–525/7.5 mL), and total number of 947 CTCs detected. Of the 22 patients with RareCyte enumeration data, 18 patients also had matched CellSearch counts. Among these 18 patients with both RareCyte and CellSearch data, CTCs were detected in 11 patients (61%) by CellSearch (median 3 CTCs/7.5 mL, range 1–343/7.5 mL), and in 15 patients (83%) by RareCyte (median 1.5 CTCs/7.5 mL, range 1–525/7.5 mL). Within each patient, RareCyte and CellSearch CTC counts were highly concordant (R^2^ = 0.88, Figure 2A).

A second RareCyte blood tube was collected from each patient for quantitative immunofluorescence characterization of either Arv7 alone or in combination with SYP (Figure 2B). A total of 284 CTCs from 12 patients were analyzed; ≥1 Arv7 + CTC was identified in 4 patients (33%), ≥1 SYP + CTCs identified in 5 patients (42%), with 3 patients having both Arv7+ and SYP + CTCs. Arv7, cytokeratin (CK) and SYP MFI distribution were highly variable within each subject’s individual CTCs indicating a great deal of intrapatient heterogeneity (Figure 2C,D). Notably, in the 284 CTCs analyzed, Arv7 and SYP appear to be mutually exclusive with only 1 CTC positive for both (Figure 2E).

### 2.3. Matched CTC and cfDNA Targeted Amplicon Sequencing

Cell-free DNA concentrations in the plasma isolated from RareCyte BCT ranged from 0.5 to 223.6 ng/mL plasma (average 17.5 ng/mL), and cfDNA concentrations in the plasma from Streck DNA tubes ranged from 0.2 to 186 ng/mL plasma (average 18.7 ng/mL plasma). There were no significant differences in cfDNA concentrations observed in individual patients across blood collection tubes (Table S2). Using RareCyte’s integrated Cytepicker module, individual CTCs were successfully recovered from 15 subjects with CTCs (range 1–4 CTCs recovered per sample). In total, 37 individual CTCs and 30 white blood cells (WBCs) as germline controls were isolated. All CTC and WBC single-cell DNA (scDNA) samples, matching cfDNA (DNA Streck and RareCyte BCTs), and buffy coat DNA (bcDNA) underwent library generation using a Custom Ion AmpliSeq™ HD assay replicating the content of the Oncomine™ Pan-Cancer Cell-Free Assay covering over 800 COSMIC mutations and copy number alterations in 52 cancer-related genes. Targeted amplicon sequencing was performed for all CTCs and cfDNA libraries provided the ratio of primer dimer to library concentration (pM) was ≤0.5, resulting in the removal of one patient’s matched cfDNA samples. 

**Figure 3:**
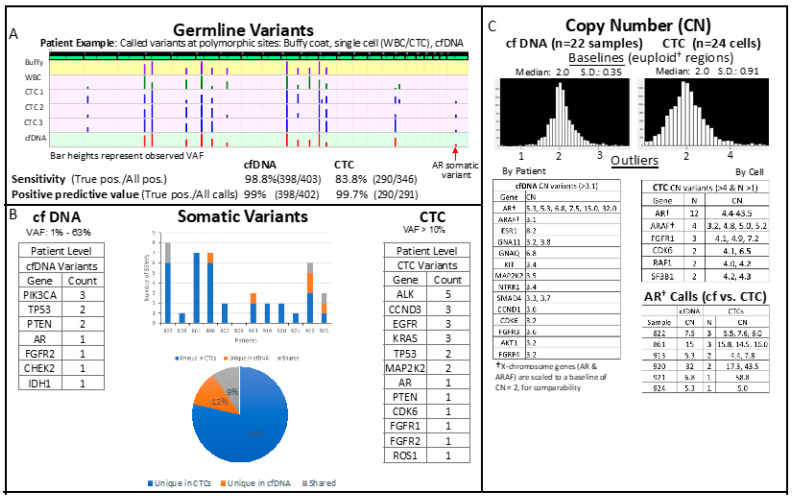
Summary sequencing results of liquid biopsy samples. (**A**). Variant calling sensitivity and positive predictive value estimated using germline variant detection in cfDNA and CTCs. (**B**). Detection of somatic variants in cfDNA and CTC across patient cohort. (**C**). Distribution of gene-level copy number (CN) estimates in cfDNA and CTCs, with copy number variants tabulated separately. Androgen Receptor CN estimates show high consistency, both between cfDNA and CTC analysis, and between CTCs (bottom table).

For germline variants, where ground truth can be determined (from WBC and buffy coat data), cfDNA yielded high sensitivity (98.8%) and positive predictive value (PPV, 99%). A subset of CTCs (11 of 35) had high counts of false negatives of germline variants (more than 4), and we chose to filter out these poor performers; the remaining 24 CTCs (from 13 patients) showed high PPV (99.7%) (Figure 3A). Note that sensitivity calculation is based in part on the false-negative count and is thus not meaningful for CTCs when cells have been filtered based on this count.

Somatic single-nucleotide variants (SSNVs) were detected in cfDNA in 10 out of 22 patients (45%). Based on LOD values calculated for each sequence variant, cfDNA allele frequencies as low as 0.1% were called. SSNVs were detected in CTCs in 12 out of the 13 patients with high quality sequencing data (92%; MAF > 10%). In cfDNA and in CTCs, variants were identified in common prostate cancer-related genes including *AR*, *TP53*, *PIK3CA*, *ALK* and *PTEN* (Figure 3B). A total of 16 SSNVs were detected across all patients cfDNA; whereas 38 SSNVs were identified across all patients’ CTCs. The number of somatic variants observed across all patients’ CTCs ranged from 0 to 11, with the majority of variants identified in only a single CTC, possibly indicating a high level of heterogeneity between CTC variants (albeit in a small number of patients). Similarly, while some mutations were concordant between subject matched CTC scDNA and cfDNA, most were distinct (Figure 3B). 

Copy number alterations were called in cfDNA in 12 out of 22 patients and called in 12 out of 13 patients with CTCs passing QC, with copy number gain/amplifications identified in common prostate-related genes, the most prominent being *AR* followed by *ARAF*, *FGFR1* and *CDK6*. *AR* copy number gains were identified in both cfDNA (range 5.3–32.0 copies) and CTCs (4.4–43.5 copies) in six patients, with high consistency demonstrated between CN calls between subject matched cfDNA and CTCs and between CTC to CTC (Figure 3C). 

### 2.4. Cluster Analysis

**Figure 4:**
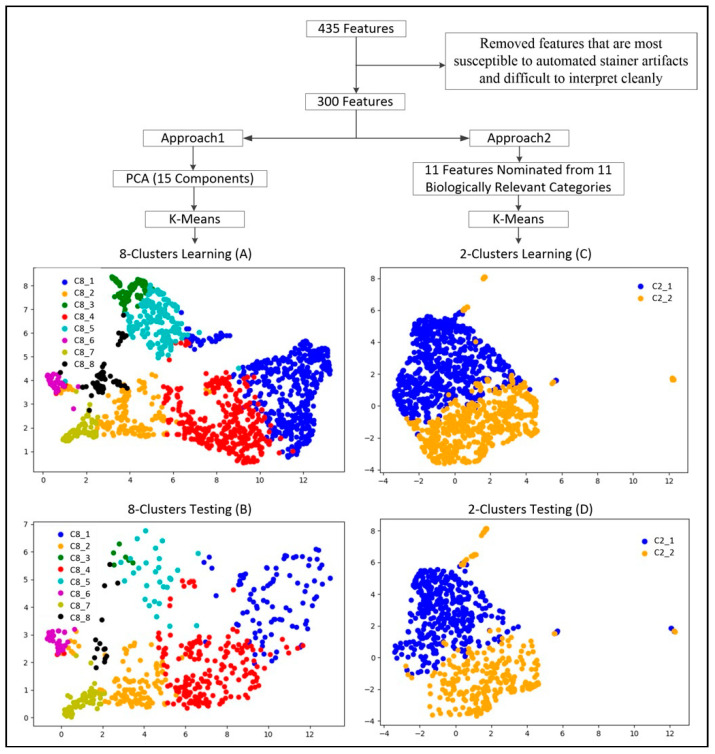
U-Map from K-Mean Clustering. **Top**: Feature selection process and number of inputs for the two K-means cluster analytic approaches (PCA vs. features nomination). **Bottom**: U-maps represent the clusters discovered from machine learning. U-maps A&C are from training data, B&D are from independent testing data. The training and testing U-maps shared similar patterns in cluster distribution.

Unsupervised machine learning techniques were applied to group the observed CTCs into clusters based on immunofluorescence and morphological features captured by the RareCyte platform in order to relate the clusters to clinically relevant phenotypes. Two approaches to clustering were applied to the data. The first approach, using a PCA reduced feature set, resulted in 8 clusters as the most frequently determined number of clusters among the 400 iterations (86/400). The second approach used single features nominated from 11 categories that capture a mixture of nuclear, cytokeratin and cellular shape and texture features known to be useful for categorizing CTCs [34,35]. Clustering models trained with these features using the gap method approach resulted in the selection of 2 cluster models for each of the 30 iterations. The final clusters from the learning and independent testing data for both approaches were illustrated in Figure 4 using U-map projection of the training data to reduce the high-dimensional data to 2D for visualization. The patterns and distribution of the clusters in the independent test set closely mirror the training set and all subsequent analysis was performed only using the test set of CTCs.

**Figure 5:**
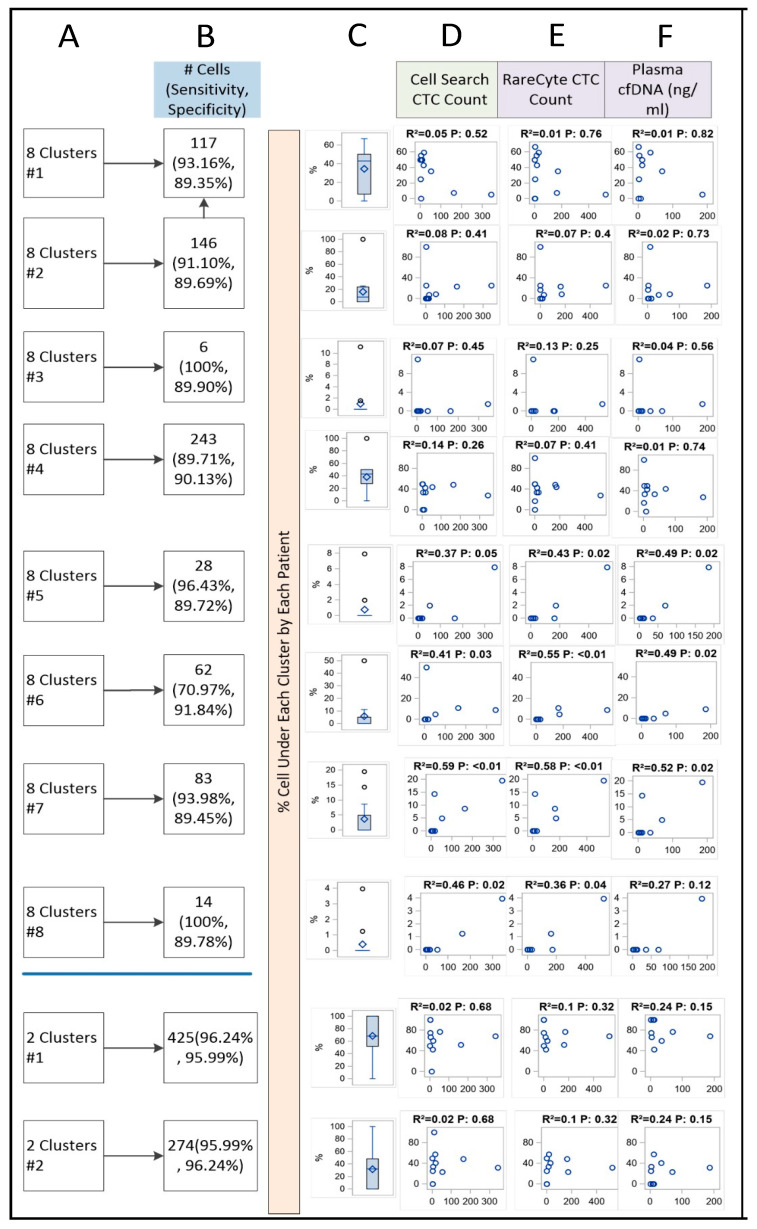
Correlation between Cell Cluster Concentration, CTC Count, and cfDNA. Column (**A**): Cluster label. Column (**B**): Number of CTCs and prediction accuracy (sensitivity/specificity) when using morphological features to predict clusters identified by K-means machine learning. Column (**C**): Distribution of percent CTC from each patient that fell into a given cluster. Column (**D**–**F**): Correlation between percentage of cell count under a given cluster within an individual and total CTC numbers and cfDNA level at the patient level.

Unpacking Clusters: The CART results of unpacking the eight clusters and two clusters using independent testing data are shown in Figure 5 and Supplementary Table S3. Cross-validation results showed that the majority of the clusters reached >80% accuracy when using individual features directly to predict cluster assignment for individual CTCs, in either the 8 cluster or the 2 cluster models (Figure 5; columns A, B).

Correlation with CTC count: At the patient level, clusters 5, 6, 7 and 8 showed strong correlation with CTC counts as well as plasma cfDNA with R^2^ ranging from 0.24 to 0.59. That is, for a given patient, a higher percentage of CTCs in one of those clusters was statistically significantly associated with higher total CTC numbers and cfDNA levels. The scatter plot for each pair of correlation was illustrated in Figure 5 (columns D, E, F).

### 2.5. Association between Radiomics and Liquid Biopsy Readouts

**Figure 6:**
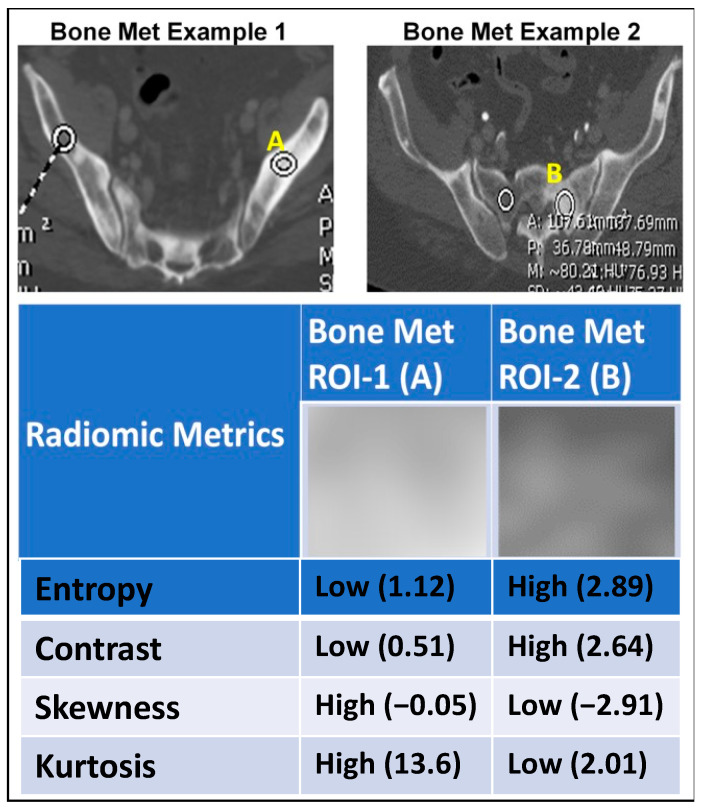
Visual representation of select radiomic metrics. Sample structured report with values of the intensity characterization metrics, namely entropy, contrast, skewness and kurtosis, obtained across two different bone metastatic lesion from two different patients.

**Figure 7:**
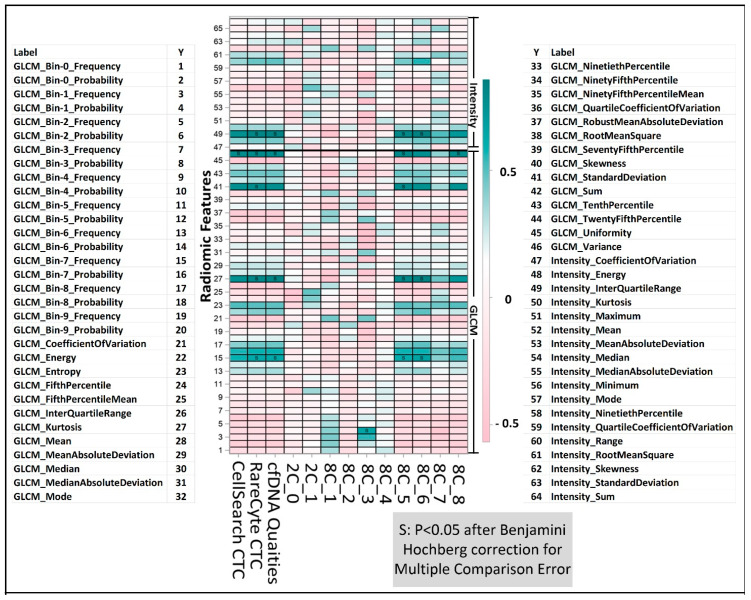
Liquid biopsy correlation with radiomic features. Heatmap representation of the correlation between radiomic panel features and liquid biopsy readouts. Here, c2_0 and c2_1 represent percent of cells belonging to the first or the second cluster of the 2-cluster analysis. c8_1 through c8_8 represent the percent of cells belonging to each of the 8 clusters of the 8-cluster analysis. Color bar depicts the correlation coefficient, a positive number (green) means positive correlation, and a negative number (pink) means negative correlation. Intensity: Signal Intensity Metrics, GLCM: Grey Level Co-Occurrence Matrix.

Radiomics-based texture analysis in CT scans of metastatic bone lesions of prostate cancer was performed with CaPTk software. The ROIs for the texture analysis were defined by manual segmentation using the ITK software. To avoid overfitting, radiomic analysis was focused on intensity characterization and grey-level co-occurrence matrix analysis. As an example, Figure 6 shows a structured report with sample values of the intensity characterization metrics, namely entropy, contrast, skewness and kurtosis, with associated texture metrics, obtained across two different bone metastatic lesion from two different patients, representing the outer bounds of entropy within our patient cohort. While the visual appearance of the two ROIs is often similar, their quantitative characterization via texture metrics helps objectively differentiate the two cases. The heatmap in Figure 7 depicts the pattern of association between each radiomic and molecular feature. Intensity interquartile range, GLCM variance, GLCM standard deviation, GLCM median absolute deviation and GLCM energy have consistent and strong positive association with CTC count and plasma cfDNA as well as with CTC clusters 6, 7 and 8 discovered through K-means after PCA dimension reduction. For example, a high value of GLCM variance indicates high spatial heterogeneity in the gray levels making up the ROI. Here, we show that patients with high radiomic heterogeneity have associated high CTC counts, plasma cfDNA levels, and CTC morphometries, as represented by unique clusters.

### 2.6. Sample Patient

**Figure 8:**
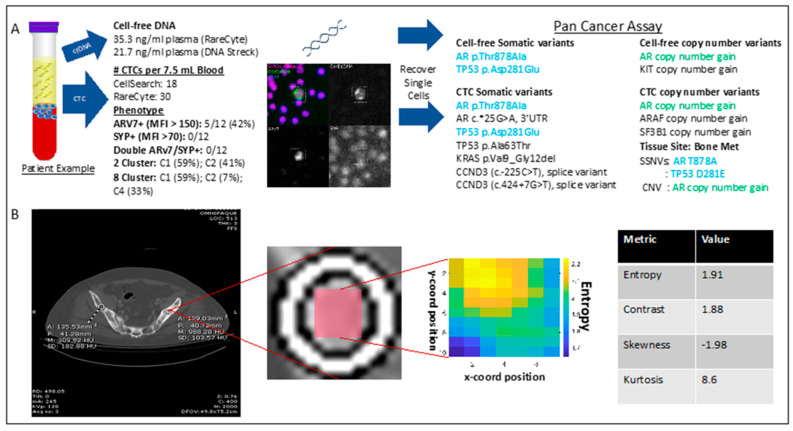
Multiparametric profile of individual patient. (**A**) Liquid biopsy result summary for an individual mPC patient (ID number 822). Results displayed include cell-free DNA quantity, CTC counts and cellular phenotypes (**left**), and detection of somatic variants in cfDNA, CTC and tissue biopsy (**right**). Blue indicates somatic variants shared in cfDNA, CTC and tissue biopsy. Green indicates copy number variants shared in cfDNA, CTC and tissue biopsy. (**B**) Radiomic analysis performed on a metastatic bone lesion (round ROI). Lesions were identified and segmented by an expert radiologist using ITK software. In all cases, the normal bone (square ROI) was also segmented. From within the segmented contours, the largest ROIs (shaded in red) were extracted for radiomics analysis. Texture analysis was performed with CaPTk software. To avoid overfitting in this pilot study, radiomic analysis was focused on intensity characterization and grey-level co-occurrence matrix (GLCM) analysis.

An integrated multi-parametric molecular and phenotypic profile was generated using the liquid biopsy and radiomic workflows described above (Figure 8). A 71-year-old man with mCRPC, previously treated with androgen deprivation therapy (ADT), abiraterone, enzalutamide, sipuleucel-T, radium 223 and docetaxel, experienced disease progression (PSA increase from 365 ng/mL to 523 ng/mL), at which point a blood sample was drawn for this study. Initial analysis of the liquid biopsy components revealed high concentrations of cfDNA (35.3 ng/mL plasma) and high CTC counts on both the CellSearch and RareCyte platforms, with 18 CTCs/7.5 mL and 30 CTCs/7.5 mL identified, respectively. Further CTC phenotypic analysis on the RareCyte platform revealed that ~30% of CTCs were Arv7 positive, with Arv7 CTCs showing a wide range of MFI values. No SYP-positive CTCs indicative of a neuroendocrine phenotype were identified. Three individual CTCs were analyzed for SSNVs and were found to have somatic variants in *AR* (all CTCs), *TP53* (all CTCs), *KRAS* (1 CTC) and *CCND3* (1 CTC). The two most common alterations in TP53 and AR were found in matched plasma cfDNA (both RareCyte and DNA Streck BCT) samples. Interestingly, these same two somatic variants (*AR* T878A and *TP53* D281E) were concordant with solid tumor NGS results from a bone metastatic biopsy six months previous. Additionally, remarkable consistency in copy number calls for *AR* amplification were identified across both liquid biopsies cfDNA (*n* = 7.5) and CTCs (*n* = 5.5, 6.0, 7.6 copies) and solid tumor NGS. Additional amplifications were found in other cancer-related genes including *ARAF*, *SF3B1* in CTCs and *KIT* in cfDNA. Radiomic analysis revealed the entropy, contrast, skewness and kurtosis of this patient’s bone metastases were 1.91, 1.88, −1.98 and 8.6, respectively, which are considered high for entropy, contrast and kurtosis and, low for skewness when referencing to the distribution from the entire study sample. Higher values of entropy, contrast and kurtosis and lower values of skewness are associated with high heterogeneity in gray levels in this ROI and constitute a feature pattern that is associated with high CTC counts and cfDNA levels (Figure 7).

## 3. Discussion

In this study, we have demonstrated the feasibility of a streamlined, multi-parametric, non-invasive profiling approach that integrates liquid biopsy readouts for CTC enumeration, CTC Arv7 and SYP staining, SSNVs and CNVs from both CTC and cfDNA, and CTC clustering analysis together with comprehensive radiomic analysis of bone metastasis from CT scans. We showed that the liquid biopsy readouts described could be obtained using RareCyte’s CTC characterization technology and an identical sequencing panel to allow for direct comparison of cfDNA and CTC variants. In addition, we found that non-invasive CT-based radiomic analysis from standard-of-care imaging was feasible, providing an additional layer of patient level information that could be correlated with liquid biopsy analysis. The mCRPC patient cohort in this study was limited in size and treated with heterogeneous therapies. Therefore, correlation of multi-parametric profiles with clinical endpoints such as disease progression or overall survival was beyond the current project scope. Rather, our purpose was to demonstrate the feasibility of this approach and the additional layers of complementary data to be gained, as shown in the sample patient (Figure 8). As a next step, we are currently validating multi-parametric profile correlations with clinical outcomes in advanced prostate cancer as part of an ongoing phase 3 multi-center trial.

The utility of CellSearch CTC counts as a clinically validated prognostic biomarker in both metastatic hormone-sensitive and castration-resistant prostate cancer is well established [9,10,11]. Here, we compared the CTC detection rate of CellSearch and RareCyte platforms in a cohort of men with advanced prostate cancer that had received a median number of 4 lines of therapy and in whom 60% had evidence of progressive disease, thus increasing the likelihood of identifying CTCs. We found that RareCyte identified a few more patients with CTCs (15 vs. 11) than CellSearch, but the four patients identified only by RareCyte had very low CTCs (usually 1 CTC/7.5 mL). Among the 11 patients that were CTC+ on both RareCyte and CellSearch, the CTC counts were highly concordant between the two platforms (R^2^ = 0.88).

To extend the potentially clinically relevant cellular readout beyond counts alone, we also stained CTCs for two prostate cancer-relevant markers that imply treatment resistance to ARSIs: Arv7 and SYP. SYP is a classical neuroendocrine marker, that is expressed in neuroendocrine prostate cancer, an aggressive subtype of prostate cancer. Patients can present with de novo neuroendocrine prostate cancer at low frequencies (<2%), with enrichment of this subtype (10–20%) occurring upon treatment of CRPC with ARSIs because of treatment resistance [35,36]. Likewise, Arv7, a constitutively active androgen receptor splice variant lacking the ligand-binding domain, is present at low frequencies in de novo prostate cancer, but frequency increases upon treatment with ARSIs. Studies confirmed that mCRPC patients with CTCs expressing Arv7 had poorer PFS and OS when treated with ARSI when compared to standard chemotherapies [20,21,37]. Here, we found that half of our patients with identifiable CTCs were positive for either Arv7 or SYP. The high prevalence of positivity identified in these patients versus other larger controlled clinical trials likely owes to the heavily pretreated nature of our cohort, with all positive patients having been treated with at least two lines of ARSI. We identified individual patients with CTCs that were positive for Arv7, SYP, or negative for both markers. This phenotypic heterogeneity mirrors the finding from the recent PROPHECY study where mCRPC patients treated with ARSIs were queried for CTC Arv7, chromosomal instability and neuroendocrine phenotype by immunomorphology. Patients were found to have different combinations of the CTC biomarkers, which each independently associated with worse overall survival [35]. Interestingly, in our cohort, Arv7- and SYP-positive CTCs appear to be mutually exclusive, likely representing two independent strategies employed by prostate cancer cells to achieve resistance to ARSI. 

Studies mapping the cellular and molecular landscape of PC have identified phenotypes that are associated with treatment resistance and disease progression [38,39,40,41,42,43,44,45,46,47,48,49,50,51,52,53,54,55]. CTCs and ctDNA genomic data can be analyzed non-invasively and repeatedly throughout disease course, illuminating how tumor phenotypes change in real time during treatment, and how such changes ultimately impact clinical outcomes. However, to date, few studies have applied the same sequencing techniques to matched CTCs and cfDNA from the same blood samples, a critical step to determine how these assays perform on single-cell vs. cell-free starting material, what information they yield, and whether that information is concordant or complementary. In this feasibility study, we successfully generated genomic profiles comprised of matched single CTCs and ctDNA SSNVs and copy number alterations from the same blood draw tube and using the same NGS-based targeted sequencing panel. *AR* copy number amplifications or gain of function mutations in the ligand binding domain commonly found in metastatic prostate cancer (~50%) were identified in our cohort. These alterations have been associated with poor prognosis and acquired resistance in mCRPC patients undergoing treatment with ARSIs [24,56,57,58,59,60,61]. In the patient example (Figure 8), our data showed high consistency across multiple CTCs, ctDNA and metastatic biopsy for both recurrent *AR* mutations and *AR* copy gain. In fact, across the entire cohort there was remarkable consistency of CN estimates in *AR* calls both between ctDNA and CTC analyses, and from CTC to CTC.

In contrast to CN, the majority of SSNVs identified were unique to either ctDNA or CTCs. The reasons for these differences are multi-faceted and may include both biological and technical variables. The ability to detect alterations in cfDNA is highly dependent on the amount of ctDNA levels in the circulation, with overall tumor burden, tumor location and timing of blood draw relative to previous treatment all important factors. This would imply that the reason cfDNA analysis resulted in fewer identified alterations was a sensitivity issue. If we had sequenced to greater depth, perhaps we would have detected an equal number of ctDNA alterations as identified in the matched CTC samples. Conversely, some alterations identified in CTCs could be a result of the greater level of amplification needed to generate sequencing libraries from ultra-low (single-cell) input DNA, which may generate false positives. Moreover, analyzing one or two CTCs per patient (as done here due to cost constraints) may not suffice, because some scDNA libraries may not pass QC and be filtered from the analysis as occurred in our study. Ideally, as more efficient and cost-effective workflows are adopted, multiple CTCs would be recovered and sequenced from each patient, yielding more robust and complete scDNA profiles. In addition to these technical factors, there are biological differences in how CTCs or ctDNA are shed into the circulation, a process that is still not fully understood. ctDNA is either actively shed or released from dying or necrotic cells, often as a response to treatment, whereas CTCs are released into the blood circulation either as single cells or as CTC clusters and can represent a heterogeneous population. These biological factors may also contribute to the differences in SSNVs identified. Notably, our findings are consistent with other studies in prostate cancer and other cancers that indicate that both CTCs and ctDNA can offer real-time complementary predictive information [17,62,63]. In recent years, the liquid biopsy field has gravitated towards analyzing ctDNA alone for genomic alterations due to relative ease of collection and analysis, ability to profile all patients, and access to genomic information pooled across multiple metastatic lesions. However, ctDNA profiling has certain shortcomings that CTCs may help to address. For example, as just noted, analysis of CTC DNA may yield additional clinically relevant genomic alterations, as found in our study and by others. Additionally, CTC analysis provides single-cell resolution that allows for assessment of overall tumor heterogeneity, associated with poor prognosis in mCRPC. Likewise, single-cell resolution provides an ability to identify and track emerging treatment-resistant clones. Finally, having the ability to correlate CTC genomic information with other protein, methylation and transcriptomic readouts at a single-cell level may provide novel insights into mechanisms of metastasis and therapy resistance.

Another layer to CTC characterization is the digital extraction of CTC morphological features combined with machine learning algorithms to develop novel CTC profiles. In advanced prostate cancer, CTC biomarkers of phenotypic heterogeneity and chromosomal instability have been developed that can identify mCRPC patients with poor prognosis when treated with ARSIs [34,35,64]. Clinical validation of the latter as a surrogate for homologous recombination deficiency is currently ongoing in a phase 2 trial in men with mCRPC treated with a PARPi (Clinical trials.gov: NCT03712930). Here, we developed the methodology and machine learning algorithms to identify clusters of CTCs that were robustly and reproducibly validated in two independent CTC datasets. Interestingly, using these techniques, we identified correlations between presence of certain CTC clusters and levels of cfDNA and CTC counts, established markers of poor prognosis. These findings hint at a possible shared biology that results both in CTCs of a certain phenotype and in high shedding of CTCs and cfDNA. While the small sample size and heterogenous nature of the patient cohort precludes us from definitively validating these correlations, it establishes a platform that enables those types of questions to be asked in large, controlled prospective clinical studies.

Radiomics represents an orthogonal and potentially synergistic approach to liquid biopsy analysis in that it is repeatable, minimally invasive and can be obtained at important treatment inflection points to inform clinical decision making. To date, studies integrating radiomic and liquid biopsy analysis are few and currently limited to pilot studies [65]. Here, methodology was developed for radiomic feature extraction from CT images of skeletal metastasis and correlations with CTC counts and cfDNA quantities were investigated. We found that high CellSearch and RareCyte CTC counts, which are established early surrogate markers of poor clinical outcome in advanced prostate cancer, had strong correlations with multiple radiomic texture-based features. Accordingly, these radiomic features could be clustered into a radiomic signature and tested for correlation with CTC counts and cfDNA levels, an earlier and more readily available surrogate endpoint than overall survival. Similarly, radiomic features may correlate not only with cfDNA levels in general but also with presence of specific genomic alterations, although the low prevalence of any one specific alteration in this small cohort precluded such an analysis.

Ultimately, integrating the cellular, molecular, and radiomic data in a multi-parametric approach will enable more comprehensive composite disease monitoring models predictive of treatment response, disease progression, and overall survival. With additional development and prospective validation, these multi-parametric biomarkers will have the potential to better inform treatment selection and improve clinical outcomes. 

## 4. Materials and Methods

### 4.1. Patient Sample Collection

All subjects gave informed consent for inclusion before they participated in this study. This study was conducted in accordance with the Declaration of Helsinki, and the protocol was approved by the University of Southern California Institutional Review Board. Blood was collected from 22 patients with mCRPC at the University of Southern California Norris Comprehensive Cancer Center who were encountered in the Norris clinics during the course of their regular care between December 2018 and February 2020. For each patient, a total of four 7.5 mL blood collection tubes (Cell-Free DNA (Streck), CellSave (CellSearch) and two RareCyte blood collection tubes (BCTs)) were collected and processed. For radiomics analysis, CT scans from this same patient cohort and an additional 22 patients with mCRPC consented previously [17] were obtained and processed.

### 4.2. AccuCyte Sample Preparation

Patient blood samples were collected in RareCyte BCTs and processed to plasma and slides using the AccuCyte^®^ Sample Preparation System [66,67]. Resulting slides containing monolayers of nucleated cells were air dried and stored at −20 °C prior to staining. Plasma collected as part of the AccuCyte workflow was placed in microcentrifuge tubes followed by centrifugation at 16,000× *g* for 10 min. The plasma supernatant was transferred to new centrifuge tubes and stored at −80 °C.

### 4.3. Immunofluorescence Staining

Slides were thawed at room temperature for 30 min, then fixed in 10% neutral buffered formalin for 40 min, followed by two washes in Tris-buffered saline (TBS) for 5 min to neutralize the formalin. Slides were stained either using the RarePlex^®^ 0900-LA CTC Panel Kit (RareCyte, Seattle, WA, USA) for CTC enumeration or using the RarePlex^®^ ARv7 CTC Panel Kit (0913-LB) and Developer Kit for synaptophysin (clone 27G12; Leica Biosystems, Wetzlar, Germany) on a Leica BOND RX automated immunohistochemistry stainer. Stained slides were coverslipped using CyteMount^®^ Mounting Medium (RareCyte) and dried at room temperature for at least one hour prior to scanning.

### 4.4. Imaging and Analysis

Slides were scanned at 10x magnification using the CyteFinder^®^ HT Instrument (RareCyte). CTC candidates were detected and rank-scored using the integrated machine-learning algorithm as described previously [67]. CTCs were visually confirmed and enumerated by a trained reviewer. In parallel, CTC enumeration was performed using the FDA-cleared CellSearch system (Menarini Silicon Biosystems, Castel Maggiore, Italy) as previously described [11].

### 4.5. Clustering Analysis

A training dataset consisting of 1482 CTCs with 435 CTC features per CTC was extracted from a cohort of breast, lung, colon, skin and prostate cancer patients (*n* = 38). The candidate features were then pared down from 435 to 300 to remove features that are most susceptible to automated stainer artifacts and those features difficult to interpret cleanly. We used two approaches for cluster analysis. The first approach started with principal component analysis. The final components were required to explain >99% of the variance of the sample; 15 components were identified. Then, K-means clustering was used with gap statistic method to identify the clusters. The range limit of cluster search was set between 2 and 50. This process was repeated 400 times. The most frequently determined number of clusters among the 400 iterations was nominated for the next step, in which we built 100 cluster models using the nominated cluster number. The model with the highest gap statistic score was selected as the final model for the first approach of cluster analysis.

An alternative approach was used to reduce dimensionality by nominating features contained within the 300 feature set that represented biologically important characteristics of CTCs. The 300 features were placed into 11 biologically relevant categories. From each category one feature was nominated by calculating the mutual information through pairwise comparisons among all feature pairs within the category using the 1482 CTC dataset and selecting the feature with the least mutual information with all other features in the category. The number of clusters was selected by the gap statistic procedure described above with 30 iterations. The final clustering model was selected by highest gap score from 50 k-means models using k nominated by the previous searching procedure. The final models from both approaches were independently validated in a separate cohort of mCRPC patients (*n* = 22), with a total number of 573 CTCs. Python scikit learn was used for all cluster analyses.

### 4.6. Interpretation of K-Means Clusters and Validation with Established Biomarker and Clinical Data

Because K-means clustering created highly condensed clusters from high dimension data, Classification and Regression Tree (CART^®^) was used to “unpack” the cluster groups. The 1482 CTC training samples were excluded from CART^®^ analysis. The Gini index was used as the splitting rule, and 10-fold cross validation was used to prune the tree. Confusion matrix from the 10-fold cross validation was used to evaluate the model performance. If the CART showed an accurate result in predicting K-means clusters, e.g., both sensitivity and specificity > 80%, the classification tree output can be used to interpret the highly condensed clusters. The Salford Predictive Modeler (8.0) from Minitab was used for CART^®^ analysis. 

At the patient level, we calculated percent CTC in each cluster from the two types of clustering methods (resulted in 8 clusters and 2 clusters, respectively), then examined the correlation with CellSearch CTC Count, RareCyte CTC Count, and plasma cfDNA (ng/mL) using Spearman’s correlation. Scatter plots was used to illustrate the correlation. SAS9.4 was used for correlation analysis.

### 4.7. Single-Cell Retrieval

Slides were incubated at 37 °C in 1X PBS to remove coverslips. De-coverslipped slides were placed in a CyteFinder Instrument (RareCyte) for semi-automated single-cell retrieval with the integrated CytePicker^®^ Retrieval Module (RareCyte). Coordinates of confirmed CTCs were imported from CyteFinder scan files, followed by mechanical retrieval as described previously [66,67]. Cells were deposited into PCR imaging tubes (RareCyte) with 1 µL picking fluid, confirmed visually, and stored at −80 °C.

### 4.8. Single-Cell Lysis

Single cells were thawed briefly, followed by addition of 6 µL Single Cell Lysis Buffer (Single Cell Lysis Kit, ThermoFisher) and room temperature incubation for 1 h. A volume of 1 µL Single Cell Stop Solution (Single Cell Lysis Kit, ThermoFisher) was added, followed by room temperature incubation for 10 min. Single-cell lysate was used as template for the Ion Torrent AmpliSeq HD Pan-Cancer Single Cell Library Prep kit.

### 4.9. Plasma cfDNA Extraction

Patient blood samples collected in Cell-free DNA BCTs (Streck) were fractionated by centrifugation at 300× *g* for 20 min. The upper layer of plasma underwent a second round of centrifugation at 5000× *g* for 10 min. Plasma cfDNA was extracted using the MagMAX™ Total Nucleic Acid Isolation Kit (Thermo Fisher Scientific, San Francisco, CA, USA) and cfDNA was quantified by Qubit™ dsDNA HS Assay Kit (Thermo Fisher Scientific, San Francisco, CA, USA) according to the manufacturer’s protocols.

### 4.10. Amplicon-Based Sequencing and Variant Calling

Libraries were generated with the Ion AmpliSeq™ HD Library Kit and custom primers covering the targets on the Oncomine™ Pan-Cancer Cell-Free Assay. Twenty-four barcoded DNA libraries generated from isolated cells were pooled and sequenced using Ion 540™ chips and the S5™ XL sequencing platform (three pools of 24 libraries in total). For plasma and buffy coat, DNA and cDNA from the same sample were combined during library construction prior to barcoding. Eight barcoded libraries were pooled and sequenced using Ion 550™ chips and the S5™ XL sequencing platform (eight pools of eight libraries in total). A single custom workflow in the Ion Reporter™ Software (v5.10) was used for variant calling. Copy number estimation was based on mean read depth across each targeted gene, adjusted for total aligned reads. Values were calculated as the ratio of observed mean depth to the median read depth across all samples.

### 4.11. Radiomic Signature or Radiomics-Based Tumor Texture Analysis

A single abdominal radiology specialist with over 15 years of experience in radiology reviewed all images. Tumor voxels belonging to 5 different non-overlapping bone metastatic lesions were segmented manually from surrounding voxels as 2D regions of interests (ROIs). ITK-SNAP (open-source software; http://www.itk.snap.org, accessed on 11 January 2021) was used for ROI segmentation [68]. The resultant ROIs from the ITK-SNAP segmentations were saved for transfer, processing and radiomic analysis. Note that typically studies perform radiomics analysis of the primary tumor, here we perform radiomics analysis of the metastatic lesions, specifically bone. Cancer Imaging Phenomics Toolkit (CaPTk) is an open-source software platform, (https://www.med.upenn.edu/cbica/captk/, accessed on 11 January 2021) that was used for feature extraction of 5 ROIs per patient [69]. While CaPTk can perform both 2D and 3D radiomic analysis, we performed only 2D radiomic analysis. All features of CaPTk are in conformance with the Image Biomarker Standardization Initiative (IBSI), unless otherwise indicated within the documentation of CaPTk [70]. The radiomic metrics featured within CaPTk quantify the characteristics of the region of the interest such as the spatial distribution of the grey levels making the region of interest (shape/size) and the complex relationship between pixels/voxels making up the region of interest (texture) using sophisticated data characterization algorithms. However, we focused on just the texture metrics; specifically, the first-order and second-order statistical metrics of texture. In general, first-order statistical metrics quantify only the intensity of the signal within a region of interest and second-order statistical metrics take into consideration the intensity as well its spatial orientation and location within a region of interest. Here, we decided to use 66 radiomic texture metrics spanning intensity characterization (*n* = 20) and grey-level co-occurrence matrix (GLCM) metrics (*n* = 46). GLCM metrics quantify the relationships between image pixels/voxels. In GLCM analysis, texture is quantified as a tabulation of how often a combination of grey-level values in an image occurs next to each other at a given distance in each direction. Additional details about the definitions of the metrics and their underlying equations can be found within the CaPTk technical reference guide (https://cbica.github.io/CaPTk/tr_FeatureExtraction.html, accessed on 11 January 2021). Post-radiomics analysis, all the extracted features across the entire patient cohort were exported in comma separated value (.csv) format for statistical analysis.

Mixed-effects models were used to examine the association between radiomic and liquid biopsy readouts, since there were 5 ROIs from each patient. The coefficient of variation was used as the covariance structure. Residual plots were used to examine model integrity. Because there were 66 radiomic features, the Benjamini–Hochberg procedure was used to control the false discovery rate inflated by multiple testing. A heat map was used to illustrate the pattern of association between radiomics features and molecular features. We have standardized each measurement to a z score before fitting the mixed-effects model; thus, the beta coefficient from the mixed-effects model can be used to approximate the correlation coefficient.

## Data Availability

The data that support the findings of this study are available from the corresponding author upon reasonable request.

## Supplementary Materials

Supplementary materials can be found at https://www.mdpi.com/article/10.3390/ijms23052571/s1.
