# Supplementary material for: Non-Invasive Profiling of Advanced Prostate Cancer via Multi-Parametric Liquid Biopsy and Radiomic Analysis
Source: Int J Mol Sci. 2022 Feb 25;23(5):2571. doi: 10.3390/ijms23052571 (PMC8910093; doi:10.3390/ijms23052571)
Supplement: Supplementary file 1 [file ijms-23-02571-s001.zip › ijms-1584012-supplementary.pdf]

Non-invasive profiling of advanced prostate cancer via multi-parametric liquid biopsy and radiomic analysis

Corresponding Author:

Amir Goldkorn, MD
Department of Medicine
NOR 3444, NRT
Health Sciences Campus
Los Angeles, CA USA
Email: agoldkor@med.usc.edu

Supplementary Tables

Supplementary Table S1. Summary chart of patient clinical data – part 1

		Mets				*CRPC Treatment History*								
**ID**	**Age**	**LN**	**Bone**	**Visceral**	**Last PSA at Blood Draw (ng/mL)**	**Abi**	**Enza**	**SipT**	**DTX**	**Other**	**No. of Therapies**	**No. of ARSI**	**BRCA Status**	**Tx at Blood Draw (R= Responding, P=Progressing)**
822	71	-	+	-	365	+	+	+	+	Radium 223, Docetaxel-taxotere	6	2	0	Lupron (P)
827	64	-	+	-	3.07	+	-	-	-	Bicalutamide, Nivolumab, Provenge	4	2	NT, mother BRCA	Abiraterone ®
828	73	+	-	-	136.8	+	+	+	+	Rucaparib	4	2	1	PARPi (P)
854	84	-	+	Lung	0.14	-	-	-	+	cabazitaxel, Cytoxan	5	1	NT	Not in charts
861	72	-	+	-	337	+	+	+	-	Radium 223, Olaparib	4	2	1, BRCA2	PARPi (P)
898	73	-	+	Lung/brain	3975	-	+	+	+	Radium 223	4	2	0	Radium 223 (P)
903	57	-	+	-	28	+	+	-	-	enzalutamide-radium 223	3	2	NT	ARSI-radium ®
908	52	-	+	-	0.63	-	-	-	+	Goserelin	1	1	NT	Docetaxel ®
909	87	+	-	Lung	42.59	-	-	-	-	Self-medication cocktail	1	1	NT	"Prosta-mix" (P)
911	72	-	+	-	8.79	-	-	-	-	Bicalutamide	1	1	NT	N/A (P)
912	82	-	-	Lung	18.66	+	-	-	+	Rucaparib	4	2	0	PARPi (R)
913	63	-	+	-	1588	+	-	-	-	Ipatasertib-abiraterone	3	1	NT	ARSI-AKT Inhibitor (P)
914	?	-	-	Liver	0	-	-	-	-	carbo-etop, carbo-irinotecan	2	0	1	Off treatment
916	64	+	-	-	1.3	+	-	-	-	N/A	2	1	0	ARSI (PSA rising/P)
917	68	?	?	?	?	?	?	?	?	?	?	?	?	?
919	71	+	-	-	0.24	-	-	-	-	-	0	0	NT, maternal history	Off-treatment
920	?	-	+	brain	27.5	+	+	?	+	radium 223, craniectomy resection	4	2	NT	ARSI-radium ®
921	?	-	+	-	24.9	+	+	?	+	?	?	?	NT	ARSI-Docetaxel (P)
922	?	-	+	-	11.1	?	?	?	+	?	?	?	NT	Docetaxel (P)
924	79	+	-	-	97.78	-	+	-	-	Bicalutamide, Apalutamide	4	3	0	Apalutamide (P)
925	72	+	+	Bladder	8.2	+	+	-	-	Bicalutamide,	4	3	NT	Radium-223 ®
928	67	+	+	Liver	9.1	+	+	-	-	Cabazitaxel, Bicalutamide	4	3	0	Cabazitaxel (P)

Supplementary Table S1. Summary chart of patient clinical data – part 2

	Solid Tumor NGS											
**ID**	**Tissue Site (Date of biopsy)**	**# SSNVs**	**# CNVs (amps/dels)**	**Additional findings**	**Mass**	**Imaging**	**Notes**	**Survival Status**	**Date of Death (If Applicable)**	**PSA At Next Visit**	**Date of "Next Visit"**	**Date of Initial Blood Draw**
822	Bone (12/27/2017)	AR T878A, TP53 D281E, TMPRSS2-ERG fusion	AR amp	MS-stable, TMB-low	CT bone mets	CT scan (11 days prior to blood draw)		Alive	NA	523.2		2/6/2019
827					CT bone mets			Alive	NA	0		12/12/2018
828	LN (12/29/2017)	BRCA2 pW993*, NOTCH1 pC1357*, RB1 c2107-2A>G	AR amp	MSS, TMB-intermediate	CT lymph mets	CT scan (15 days prior to blood draw)		Alive	NA	Not in charts		12/12/2018
854					CT bone mets	NA	Neuroendocrine features	Alive	NA	Not in charts		2/5/2019
861	Temporal lobe (12/10/2018)	BRCA2 pY1894K, pY1894*; FANCA pP1042A; RB1 c859_861+12del15		MSS, TMB-intermediate	CT bone mets	CT scan (10 days prior to blood draw)		Deceased	19-Jul	422		12/19/2018
898	Bone (02/28/2018)	PTEN C296*		MS-stable, TMB-low	CT bone mets	CT scan (14 days prior to blood draw)		Unknown / Likely Deceased	Hospice 2/27/2019	6655		2/6/2019
903					CT bone mets	CT scan (22 days prior to blood draw)		Alive	NA	147.3		2/25/2019
908	Primary (05/01/2018)	TMPRSS2-ERG	ND	MSS, TMB-low	CT bone mets	CT scan (10 days prior to blood draw)	mHSPC- ADT + Docetaxel	Alive	NA	0.03		12/12/2018
909								Alive	NA	128.9		12/19/2018
911								Alive	NA	13		1/7/2019
912				ATM mutation	lung mass mets CT	CT scan (13 days prior to blood draw)	mMIBC-chemo-RT to bladder	Alive	NA	17.98		1/9/2019
913					CT bone mets	CT scan (8 days prior to blood draw)	LAC Patient	Deceased	11/17/2019	1064	2/7/2019	1/14/2019
914	Omentum (10/09/17)	PIK3CA P134S, CTNNB1 I35_G38del, TMPRSS2-ERG, CDH1 loss exon 1-14	BRCA del	MSS, TMB-low	liver/adrenal mass CT	CT scan (5 days prior to blood draw)	Neuroendocrine features	?	?	?		1/15/2019
916					CT bone mets			Alive	NA	2.7		2/6/2019

917							LAC Patient	Alive	NA	6.84	2/28/2019	2/7/2019
919								Alive	NA	0.5		3/13/2019
920					CT bone mets	CT scan (60 days prior to blood draw)	LAC Patient	Alive	NA	27.56	3/21/2019	3/14/2019
921					CT bone mets	CT scan (2 days prior to blood draw)	LAC Patient	Deceased	10/6/2019	24.24	4/3/2019	3/14/2019
922					CT bone mets	CT scan (same day as blood draw)	LAC Patient	Deceased	6/28/2019	12.4	5/2/2019	3/14/2019
924	Blood (09/19/2018)	AR H875Y, TERT promoter - 124C>T						Unknown / Likely Deceased	After 12/30/2019	139.67		4/1/2019
925								Unknown / Likely Deceased	After 9/2019	21.4		4/10/2019
928								Alive	NA	Not in charts		5/20/2019

Supplementary Table S2. Cell-free DNA concentrations.

	RareCyte BCT	DNA Streck BCT
Patient	ng cfDNA per ml plasma	ng cfDNA per ml plasma
822	35.3	21.7
827	0.9	0.5
828	11.3	10.5
854	69.3	34.7
861	186.0	223.6
898	1.2	2.6
908	4.5	3.8
909	2.9	4.0
911	3.2	4.0
912	2.6	1.4
913	2.6	2.0
914	5.3	2.6
920	2.9	4.1
903	8.3	3.2
916	4.5	5.5
918	7.6	5.2
919	11.8	6.7
921	16.2	7.1
922	12.2	5.4
924	4.3	8.3
925	0.2	9.9
928	NT	NT

Supplementary Table S3: Clustering Classification Tree descriptors

Cluster type	Cluster number	Cluster descriptors
8 Clusters	1	ThickMembrane CkEpCAM Background Max <= 5755.71<br>WholeCell SytoxOrange StDev <= 10580.95<br>WholeCell SytoxOrange Max<= 1294.13
	2	ThickMembrane CkEpCAM Background Max <= 5755.71<br>WholeCell SytoxOrange StDev > 10580.95<br>ThickMembrane CkEpCAM Background Max <= 22226.45<br>CytoMem SytoxOrange Max <= 10253.45
	3	ThickMembrane CkEpCAM Background Max > 5755.71<br>ThickMembrane CkEpCAM Background Max <= 13469.30<br>ThinMembrane SytoxOrange StDev > 13758.95
	4	ThickMembrane CkEpCAM Background Max <= 5755.71<br>WholeCell SytoxOrange StDev <= 10580.95<br>WholeCell SytoxOrange Max > 1294.13
	5	ThickMembrane CkEpCAM Background Max > 5755.71<br>ThickMembrane CkEpCAM Background Max <= 13469.30<br>ThinMembrane SytoxOrange StDev <= 13758.95
	6	ThickMembrane CkEpCAM Background Max <= 5755.71<br>WholeCell SytoxOrange StDev > 10580.95<br>ThickMembrane CkEpCAM Background Max <= 22226.45<br>CytoMem SytoxOrange Max > 10253.45
	7	ThickMembrane CkEpCAM Background Max <= 5755.71<br>WholeCell SytoxOrange StDev > 10580.95<br>ThickMembrane CkEpCAM Background Max > 22226.45<br>Nuclear CkEpCAM Max > 2768.56
		ThickMembrane CkEpCAM Background Max <= 5755.71<br>WholeCell SytoxOrange StDev > 10580.95<br>ThickMembrane CkEpCAM Background Max > 22226.45<br>Nuclear CkEpCAM Max <= 2768.56
	8	ThickMembrane CkEpCAM Background Max > 5755.71<br>ThickMembrane CkEpCAM Background Max > 13469.30
2 Clusters	1	Nuclear Extent > 0.52

		Nuclear Extent <= 0.52<br>Nuclear SytoxOrange Entropy>2.18<br>Nuclear Eccentricity<=0.4
		Nuclear Extent <= 0.52<br>Nuclear SytoxOrange Entropy>2.18<br>Nuclear Eccentricity>0.4<br>WholeCell Eccentricity<=0.32
	2	Nuclear Extent <= 0.52<br>Nuclear SytoxOrange Entropy<=2.18
		Nuclear Extent <= 0.52<br>Nuclear SytoxOrange Entropy>2.18<br>Nuclear Eccentricity>0.4<br>WholeCell Eccentricity>0.32
